# Physiological Biomarkers of Upper Motor Neuron Dysfunction in ALS

**DOI:** 10.3390/brainsci14080760

**Published:** 2024-07-29

**Authors:** Aicee Dawn Calma, Mehdi van den Bos, Nathan Pavey, Cláudia Santos Silva, Parvathi Menon, Steve Vucic

**Affiliations:** 1 Brain and Nerve Research Center, The University of Sydney, Sydney 2139, Australiacsantosilva2@gmail.com (C.S.S.);; 2 Department of Neurosciences and Mental Health, Unidade Local de Saúde de Santa Maria, 1649-028 Lisbon, Portugal; 3 Faculdade de Medicina-Instituto de Medicina Molecular, Centro de Estudos Egas Moniz, Universidade de Lisboa, 1649-028 Lisbon, Portugal

**Keywords:** cortical hyperexcitability, motor neuron disease, amyotrophic lateral sclerosis, short interval intracortical inhibition, transcranial magnetic stimulation

## Abstract

Upper motor neuron (UMN) dysfunction is an important feature of amyotrophic lateral sclerosis (ALS) for the diagnosis and understanding of pathogenesis. The identification of UMN signs forms the basis of ALS diagnosis, although may be difficult to discern, especially in the setting of severe muscle weakness. Transcranial magnetic stimulation (TMS) techniques have yielded objective physiological biomarkers of UMN dysfunction in ALS, enabling the interrogation of cortical and subcortical neuronal networks with diagnostic, pathophysiological, and prognostic implications. Transcranial magnetic stimulation techniques have provided pertinent pathogenic insights and yielded novel diagnostic and prognostic biomarkers. Cortical hyperexcitability, as heralded by a reduction in short interval intracortical inhibition (SICI) and an increase in short interval intracortical facilitation (SICF), has been associated with lower motor neuron degeneration, patterns of disease evolution, as well as the development of specific ALS clinical features including the split hand phenomenon. Reduction in SICI has also emerged as a potential diagnostic aid in ALS. More recently, physiological distinct inhibitory and facilitatory cortical interneuronal circuits have been identified, which have been shown to contribute to ALS pathogenesis. The triple stimulation technique (TST) was shown to enhance the diagnostic utility of conventional TMS measures in detecting UMN dysfunction. Resting-state EEG is a novel neurophysiological technique developed for directly interrogating cortical neuronal networks in ALS, that have yielded potentially useful physiological biomarkers of UMN dysfunction. The present review discusses physiological biomarkers of UMN dysfunction in ALS, encompassing conventional and novel TMS techniques developed to interrogate the functional integrity of the corticomotoneuronal system, focusing on pathogenic, diagnostic, and prognostic utility.

## 1. Introduction

Amyotrophic lateral sclerosis (ALS) is a rapidly progressive and fatal neurodegenerative disorder of the human motor nervous system, characterised by dysfunction of upper (UMN) and lower motor neurons (LMN) [1,2,3,4]. The focal onset of disease in the limbs is typical for ALS, with progression to involvement of multiple body regions including bulbar and respiratory regions, and eventually global muscle wasting and weakness, with respiratory dysfunction representing the terminal phase of ALS [4,5]. The concomitant dysfunction of upper and lower motor neurons has formed the basis of ALS diagnosis [6,7,8], and is pertinent to the understanding of disease pathogenesis. A long-standing debate in ALS relates to site of disease onset [9], with three main hypotheses proposed: (i) *cortical onset*, whereby descending corticomotoneuronal tracts mediate LMN degeneration through an anterograde glutamatergic excitotoxic mechanism [10,11], (ii) *LMN onset* with secondary UMN dysfunction [12,13,14,15], and (iii) *independent degeneration* of the upper and lower motor neurons evolving in a contiguous and random pattern [16,17]. Understanding the relationship between upper and lower motor dysfunction would be important for understanding ALS pathophysiology.

The emergence of physiological biomarkers of upper and lower motor neuron dysfunction has provided novel insights into ALS pathogenesis and led to the development of potentially useful diagnostic and prognostic biomarkers. Consequently, approaches to clinical trial designs have emerged, enabling patient stratification and monitoring of outcomes, as well as identifying novel therapeutic targets and treatment strategies. The present review will discuss the utility of physiological biomarkers of UMN dysfunction, focusing on recently developed transcranial magnetic stimulation (TMS) techniques, with an emphasis on pathogenic, diagnostic, and prognostic utility in ALS.

## 2. Transcranial Magnetic Stimulation

Transcranial magnetic stimulation (TMS) is a non-invasive and relatively painless neurophysiological technique enabling the assessment of UMN function in a clinical setting [18]. The technique was first described in the 1980s [19], and since then, refinement of TMS methodology and stimulus paradigms has enabled a more robust and clinically meaningful interrogation of UMN physiological in ALS.

**Figure 1:**
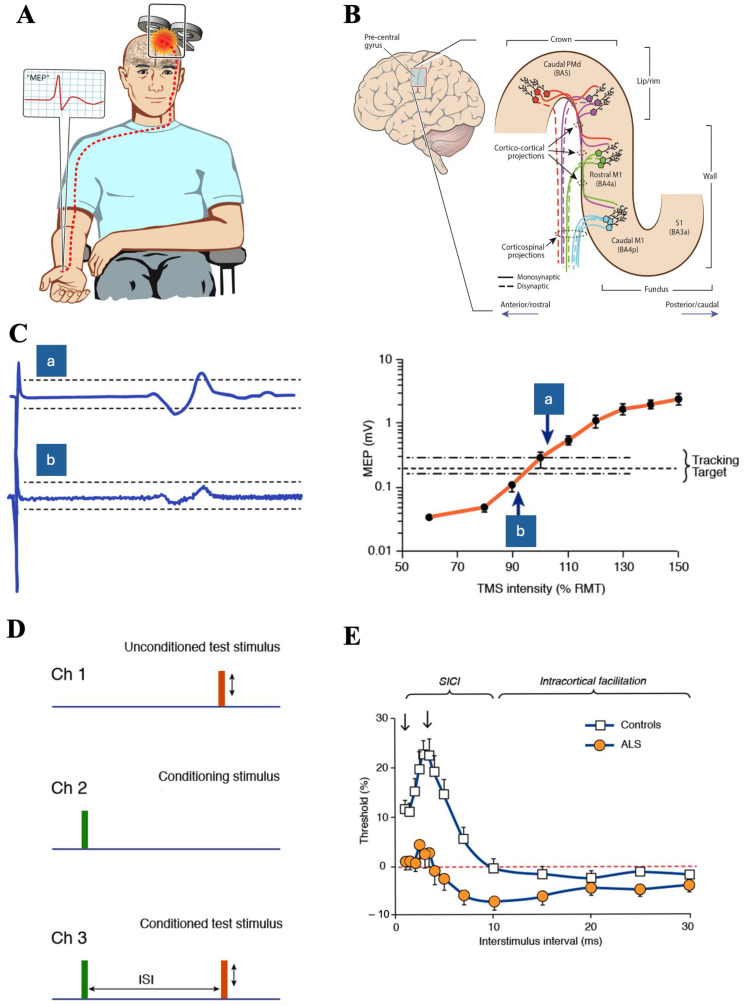
(**A**) Transcranial magnetic stimulation using a figure of eight-coil applied over the primary motor cortex (M1) elicits a motor-evoked potential (MEP, red potential in inset) from a target skeletal muscle. (**B**) Descending corticomotoneuronal pathways from the precentral gyrus contribute to the MEP responses. Direct neuronal activation most likely occurs in the lip/rim regions of the motor hand knob. There is both rostral and caudal spread of activation at the M1, through cortico-cortical synaptic neurotransmission. (**C**) Stimulus–response curve. For threshold tracking TMS, a target of 0.2 mV (±20%) is selected which lies in the steepest part of the stimulus–response curve. As such, if the MEP response is smaller than the tracking target (potential b), the subsequent stimulus is increased, whereas if the MEP response is larger than the tracking target, the subsequent stimulus intensity is decreased (potential a). (**D**) Paired pulse threshold tracking paradigm. Channel 1 records an unconditioned test stimulus, defined as the TMS intensity needed to elicit and maintain the tracking target, representing the resting motor threshold (RMT) when applying the threshold tracking technique. Channel 2 monitors the subthreshold conditioning stimulus (which does not generate MEP). Channel 3 records the conditioned test stimulus at interstimulus intervals (ISI) between 1 and 30 ms. (**E**) When using the threshold tracking TMS technique, short interval intracortical inhibition (SICI) is defined as the amount of increase in the conditioned-test stimulus intensity required to generate and maintain the tracking target, developing between ISIs of 1–7 ms. Intracortical facilitation is defined as reduced conditioned-test stimulus intensity, developing between ISIs 10–30 ms. In ALS patients, cortical hyperexcitability is reflected by reduction in SICI and an increase in ICF. Adapted with permission from Ref. [3].

The TMS stimulator delivers a brief but large current through a coil positioned over the scalp, generating a strong time-varying electromagnetic field (with a peak field up to ~2 Tesla) perpendicular to the coil [20]. The magnetic field induces an electric field in the underlying motor cortex, preferentially stimulating intracortical interneurons that in turn trans-synaptically activate pyramidal neurons (Figure 1A,B) [3,20]. Corticospinal axons may be directly activated with higher TMS intensities. A motor-evoked potential (MEP) is recorded from a target muscle in the limbs and reflects the activation of spinal motor neurons by descending corticospinal volleys (Figure 1B). Multiple factors determine the distinct neuronal elements that are stimulated with each pulse. These factors include coil shape and field depth, the direction of induced cortical currents, pulse waveform, the strength of stimulation, and the number of delivered TMS pulses [20,21,22,23,24,25,26,27].

TMS is a now well-established neurophysiological technique for assessing UMN in ALS, leading to a greater understanding of pathophysiology and the development of novel diagnostic and efficacy biomarkers [3,28,29]. Novel physiological biomarkers of UMN dysfunction in ALS have been reported utilising single, paired, and triple pulse TMS techniques, as well as TMS-EEG and resting-state EEG, and their clinical applicability as pathogenic, diagnostic, and prognostic biomarkers will be discussed.

## 3. Pathophysiological Biomarkers of UMN Dysfunction

*Paired-pulse TMS*: Paired-pulse TMS techniques deliver two consecutive stimuli at varying interstimulus intervals (ISI), with the conditioning stimulus modulating the effects of a subsequent test stimulus. The paired-pulse TMS method has proven invaluable for interrogating the importance of interneuronal circuit dysfunction in ALS pathogenesis, heralded novel UMN diagnostic biomarkers that have been integrated into clinical trial designs. Short interval intracortical inhibition (SICI), short interval intracortical facilitation (SICF), intracortical facilitation (ICF) and long interval intracortical inhibition (LICI) have been utilised in ALS [3,18,23], with SICI exhibiting greatest utility [30].

*Short interval intracortical inhibition was* first described in 1993 and is a sensitive biomarker of motor cortical excitability, reflecting the activity of inhibitory interneuronal circuits acting via GABA_A_ receptors [31,32]. SICI is elicited when a suprathreshold test stimulus (TS) is preceded by a subthreshold conditioning stimulus (CS) at inter-stimulus intervals (ISI) of 1-to-7 ms. In the conventional “constant stimulus” method, the CS and TS are kept at a constant intensity, and SICI is reflected by a reduction in the conditioned TS-induced MEP amplitude compared to the unconditioned TS [31]. Subsequently, the threshold tracking paired-pulse TMS technique (TT-TMS) was developed (Figure 1), whereby changes in TS intensity required to maintain a target MEP response, fixed at an amplitude of 0.2 mV (±20%), are tracked at ISIs that are increased in a sequential ascending [33,34] or parallel order [35]. In the TT-TMS paradigm, SICI is reflected by a higher conditioned-test stimulus intensity required to generate and maintain the target MEP response between ISIs of 1-to-7 ms [33,34] (Figure 1E). The reliability and reproducibility of mean SICI, as measured by the serial ascending method, have been established in healthy controls [36]. A U-shaped curve relationship was established between SICI and CS intensities [37,38,39,40,41], suggesting that SICI may also reflect a balance between inhibition and facilitation. More recently, physiologically distinct interneuronal circuits with variable cortical orientation and thresholds were reported to contribute to SICI [27].

Reduction or absence of SICI has been consistently identified in ALS, irrespective of the used methodology [28,42,43,44,45,46,47,48,49,50,51,52,53,54,55,56]. The reduction in SICI correlates with and may precede LMN dysfunction in both sporadic and familial ALS phenotypes [28,43,48,57,58]. Abnormalities of SICI may be evident focally within the motor cortex, mirroring disease onset [59]. Reduction in SICI is also associated with patterns of disease progression [55,59,60] and shorter survival [61]. Separately, SICI reduction has also been reported in familial ALS phenotypes linked to mutations in the superoxide dismutase-1 [51], fused in sarcoma [62] and c9orf72 genes [58]. Asymptomatic mutation carriers exhibit normal cortical function [51,58], with SICI reduction preceding clinical development of familial ALS by months [51], thereby suggesting that epigenetic and environmental factors are important in initiating the disease. Such a notion is in keeping with the multi-step hypothesis of ALS [63,64,65].

Short-interval intracortical inhibition reduction has been associated with the split hand and split hand-plus signs [66,67], both recognised clinical features of ALS [68,69,70,71]. More prominent SICI reduction was reported when recording over the lateral hand groups of muscles (abductor pollicis brevis [APB] and first dorsal interosseous) compared to hypothenar or flexor pollicis longus muscles [66,67]. At a peripheral level, axonal excitability changes did not follow a split-hand pattern of distribution, suggesting a cortical contribution to the split-hand phenomenon. It should be stressed that peripheral factors encompassing neuromuscular junction dysfunction [72], as well as an increase in excitability of axons innervating the APB muscle [73], have also been shown to contribute to the split hand phenomenon.

The abnormalities of cortical inhibition have also been reported in atypical ALS phenotypes, including the flail arm and leg variants of ALS [56,74,75], which correlated with neurodegeneration and thereby underscored the pathogenic significance. Recently, the parallel threshold tracking paradigm also disclosed a more prominent reduction in SICI in ALS patients with fewer upper motor neuron signs [53,54].

It has been argued that SICI reduction may represent a compensatory mechanism in response to LMN degeneration [47]. Arguing against this notion are findings of normal cortical excitability in ALS neuromuscular mimicking disorders [28,49,76], as well as partial and transient normalisation of SICI in ALS patients treated with riluzole (anti-glutaminergic medication) [77,78]. Consequently, dysfunction of GABAergic interneuronal circuits, acting via GABA_A_ receptors, seems the most plausible explanation for SICI reduction in ALS, a notion supported by pathological studies in patients and mouse models [79,80,81]. Recently, dysfunction of distinct inhibitory interneuronal circuits, preferentially activated by TMS currents oriented in a posterior–anterior (PA) and lateral–medial (LM) direction, was reported in ALS and associated with greater cognitive deficits [82]. Given that cortical interneuronal populations express a unique combination of modifier genes, transcription factors, receptors, and neuropeptides [83], identification of these molecular processes, could provide further pathogenic insights and novel therapeutic targets. The downregulations of gene modifiers, such as LAMP5 which limits neuronal hyperexcitability in cortical inhibitory interneurons, were shown to be pathogenic in related models of neurodegenerative diseases [84]. Magnetic resonance spectroscopy (MRS) studies revealed a reduction in GABA in the ALS motor cortex [85], providing further support for inhibitory interneuronal dysfunction. In addition to reduced GABAergic function, SICI reduction in ALS could also be mediated by increased glutamatergic neurotransmission [77,78]. These findings support the hypothesis that sporadic ALS is a multistep process [64,65,86], with cortical hyperexcitability emerging as an important pathogenic step.

Abnormalities of *long interval intracortical inhibition (LICI)* have also been reported in ALS. Long interval intracortical inhibition describes the inhibition of a test MEP response that is preceded by a suprathreshold CS (50 to 300 ms) [33,87,88], and appears to be mediated by longer latency inhibitory neuronal populations acting via GABA_B_ post-synaptic receptors [32,89,90,91,92,93,94,95]. It should be stressed that LICI may inhibit SICI, probably through a pre-synaptic GABA_B_ receptor-mediated process [96].

Reduction in LICI has also been reported in ALS and is associated with a greater disease burden [47,97]. Degeneration of long-latency inhibitory circuits was proposed as a likely mechanism [32], although further studies are required to validate.

**Figure 2:**
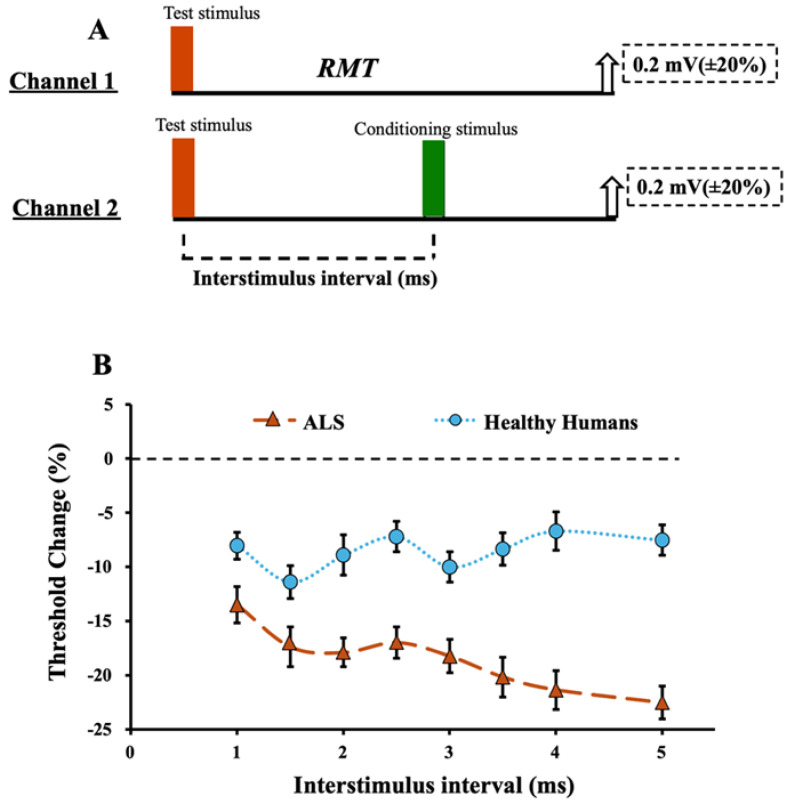
Short interval intracortical (SICF). (**A**) SICF is recorded using the paired-pulse technique using a 2-channel paradigm. On channel 1, the resting motor threshold (RMT) is defined by the intensity required to elicit and maintain a fixed target of 0.2 or 1 mV. On channel 2, a suprathreshold test response is recorded prior to a conditioning response set to 95% of the RMT. (**B**) SICF recordings in ALS and healthy controls. SICF is determined by subtracting the stimulus intensity on channel 2 from that recorded in channel 1. SICF values indicate how much less current is required to generate and maintain the tracking target of 0.2 or 1 mV. ALS subjects have more negative values indicating a higher degree of SICF.

The abnormalities of intracortical facilitatory circuits have also been reported in ALS and have been associated with pathogenesis. *Short interval intracortical facilitation (SICF)*, a biomarker of high threshold facilitatory interneuronal circuit activity, is elicited by delivering a CS set to either threshold or suprathreshold levels, ISIs of 1-to-5 ms before a TS (Figure 2A) [98,99,100]. The constant stimulus and threshold tracking methods may elicit SICF with two-three distinct peaks (Figure 2B) [98,99,100,101]. *Intracortical facilitation (ICF)* is a biomarker of longer latency facilitatory circuits that are of a lower threshold than circuits mediating SICF, and are elicited by a similar paired-pulse paradigm as SICI but at longer ISIs (8-to-30 ms) (Figure 1E) [31,33]. The physiological mechanisms remain to be fully elucidated, although the activation of low threshold excitatory glutamatergic motor cortical circuits and spinal processes have been proposed [22,98,99,102,103].

In ALS, an increase in SICF has been reported, while changes in ICF are an inconsistent finding [3,18,56,77,104,105]. The increase in SICF was accompanied by SICI reduction [105]. This imbalance in facilitatory and inhibitory neuronal function has been quantified by the *index of excitation (IE)*, which is increased in ALS and suggests overactivity of facilitatory circuits. At a pathophysiological level, IE is associated with a greater degree of functional disability and UMN signs, underscoring the importance of facilitatory circuit overactivity in ALS. As for SICI, there appears to be a proclivity for abnormality of distinct facilitatory interneuronal populations, such that SICF and IE abnormalities are only evident when stimulating the PA- and LM-oriented neuronal circuits [82]. An increase in interneuronal populations expressing the neuropeptide Y (NPY), which leads to cortical hyperexcitability, was reported in human post-mortem ALS motor cortices [106] and mouse models [107]. Consequently, the proclivity of distinct interneuronal populations to dysfunction could be explained by varied molecular properties of facilitatory cortical interneurons, although this needs further clarification. In contrast, others have reported a reduction in SICF in ALS [108]. The reason for the discordant findings is not immediately apparent and could relate to differences in patient populations and/or methods. In any case, the diagnostic utility of SICF remains to be clarified in a large ALS cohort, although when combined with SICI reduction seem to be a promising physiological biomarker [105,108].

## 4. Single Pulse TMS

*Resting motor threshold (RMT)* is a physiological biomarker of corticomotoneuronal excitability and has been traditionally assessed by the “Rossini-Rothwell” (frequency) method, defined as the lowest TMS intensity required to elicit an MEP amplitude of ≥50 µV in 50% of 10 trials within a target muscle [3,109]. With the development of the threshold tracking method, RMT is reflected by the TMS intensity required to generate and maintain a fixed MEP amplitude of 0.2 mV (±20%) [33,34]. Adaptive methodologies (maximum-likelihood or maximum a posteriori estimations) have also been used to determine RMT [23,110,111], as has a stochastic non-parametric root-finding method [111]. These newer methodologies outperform the frequency method in that they require fewer TMS stimuli and exhibit better error convergence [111]. Irrespective of the methodology used, RMT reflects the function of voltage-gated sodium channels and glutamatergic neurotransmission [112,113].

*In ALS*, changes in RMT have been heterogenous, with lower thresholds evident in the early stages of the disease, prior to the development of muscle wasting [3], and are associated with profuse fasciculations and hyper-reflexia [114,115], all indicative of corticomotoneuronal hyperexcitability [3]. The reduction in RMT is more prominent over the motor cortex contralateral to the site of disease onset [55]. Conversely, increased RMT or motor cortex inexcitability has also been reported in a minority (~10%) of ALS patients [116,117,118,119,120,121,122,123], and may be an adverse prognostic biomarker when present within the first year of symptom onset [124]. Cortical inexcitability is evident in a majority of primary lateral sclerosis (PLS) patients [74]. PLS represents a distinct neurodegenerative disorder not universally recognised as an ALS phenotype, that exhibits a markedly different progression rate and possibly different pathogenesis [125]. As the disease progresses, the corticomotoneuronal pool is slowly depleted and becomes more difficult to stimulate, leading to an increase in RMT and eventually motor cortex inexcitability [114]. RMT is significantly higher in Japanese ALS patients [30,52], a finding that could reflect the influence of racial backgrounds on RMT [52]. Overall, the discordant RMT findings in ALS may reflect the clinical and pathological heterogeneity of assessed patients. Consequently, the utility of RMT as a diagnostic physiological biomarker in ALS is limited [28,30].

*The stimulus-response (SR) curve and MEP amplitude* are mediated by the summation of descending corticospinal volleys onto the spinal and bulbar motor neurons and reflect excitability corticomotoneurons that are distinct from those mediating RMT [3,124,126]. The SR curve gradient is significantly higher in intrinsic hand muscles due to a greater density of corticomotoneuronal projections onto the motor neurons innervating these muscles [41]. Increased glutaminergic and adrenergic neurotransmission will increase, while enhanced GABAergic neurotransmission will reduce the SR gradient [32], thereby suggesting that steeper SR gradients may be a potential biomarker of cortical hyperexcitability. Moreover, the MEP amplitude is significantly smaller and more dispersed than the maximal compound muscle action potential amplitude [127], a finding related to phase cancellation and asynchronous recruitment of motor neurons secondary to the desynchronization of descending corticospinal volleys. The *MEP:CMAP ratio* is a crucial metric in clinical neurophysiology which normalises the differences in MEP amplitude that arise from individual variability in peripheral response. Additionally, it accounts for peripheral neurodegeneration, such as that seen in ALS patients, providing a more accurate reflection of central motor pathway excitability. A significant advantage of this technique is its ease of execution, making it highly feasible for implementation in clinical practise.

Marked trial-to-trial variability in MEP amplitudes has been well documented [128] and related to intrinsic fluctuations of neuronal excitability at cortical and spinal cord levels [23], as well as the timing of TMS stimulus to cortical oscillatory EEG states [129]. MEP variability can be reduced by delivering the TMS stimulus at the optimal phase of individualised β oscillation [130].

*In ALS*, steeper SR curve gradients have been, supporting enhanced glutamatergic activity and thereby the presence of corticomotor hyperexcitability [48,55,57]. Additionally, an increase in MEP amplitudes has been consistently reported as an early feature of ALS and linked to motor neuron degeneration [28,48,58,66,75,131]. Given that the MEP amplitude is significantly increased in ALS patients compared to ALS mimicking disorders argues against the notion that MEP amplitude increases represent a plasticity phenomenon in ALS [28]. The increase in SR curve gradient and MEP amplitudes is evident across physiologically distinct circuits in ALS, suggesting a contribution from multiple neuronal populations [82].

*The cortical silent period (CSP)* describes the interruption of voluntary electromyographic (EMG) activity in response to suprathreshold TMS pulse [132]. The CSP duration, measured from the onset of a facilitated MEP response to resumption of EMG activity [33], is a physiological biomarker of GABAergic neurotransmission acting via GABA_B_ receptors [133] as well as spinal inhibitory mechanisms that are mediated by activation of Renshaw cells, IA inhibitory afferents and refractoriness of spinal motor neurons [23,91,134]. Although typically elicited from the contralateral motor cortex and influenced by the density of corticomotoneuronal projections, ipsilateral silent period is well described [98]. The iSP commences 30~40 ms after the stimulus, lasting for 20–25 ms [135], and predominantly reflects transcallosal inhibition [135] with some contributions from non-callosal pathways caudal to the corpus callosum [136].

*In ALS*, a significant reduction or absence of CSP duration has been reported in early stages [47,48,49,56,58,74,75,76,131,137,138,139,140,141,142] and it is specific for ALS in the context of neuromuscular mimicking diseases, albeit exhibiting modest diagnostic utility [28,30,49,76,143]. Dysfunction of long latency cortical GABAergic inhibitory circuits, acting via GABAB_B_ receptors, appears to underlie the reduction in CSP duration in ALS, although dysfunction of spinal inhibitory circuits may also contribute. Abnormalities of ipsilateral silent period duration (i.e., absent or prolonged) have also been identified early in ALS potentially reflecting degeneration of the transcallosal glutamatergic fibres projecting onto inhibitory interneuronal circuits in the non-stimulated motor cortex [140]. A reduction in transcallosal inhibition has been reported in ALS using the threshold tracking technique [144], further supporting the dysfunction of transcallosal glutamatergic fibres in ALS.

*Central motor conduction time (CMCT)* measures conduction across the corticospinal tracts, encompassing initial activation of motor cortical neurons in the primary motor cortex through to excitation of spinal motor neurons. CMCT is measured as the difference between MEP and spinal motor neuron latencies to recording muscle, with two methods commonly used to estimate the peripheral conduction time: (i) the spinal nerve root stimulation or (ii) F-wave methods [3,18,145,146]. The conduction time is 1–1.5 ms longer when using the spinal nerve root stimulation method, due to differences in sites of peripheral nerve stimulation, and the F-wave technique is regarded as a more accurate measure of CMCT. The F-wave method can only be recorded in distal muscles and may be further limited by low F-wave persistence and high motoneuron recruitment thresholds.

*In ALS*, prolongation of CMCT has been reported and associated with corticospinal tract dysfunction [130,142]. Additionally, prolongation of CMCT may disclose subclinical UMN dysfunction in approximately 30% of ALS patients [130]. While the diagnostic utility of CMCT was suggested [130], this was not borne out in a larger study [28]. Additionally, the pathogenic significance of prolonged CMCT as a physiological biomarker seems limited given the absence of a correlation between CMCT prolongation and the degree of UMN impairment [130].

*The triple stimulation technique (TST)* is a collision method developed to increase the sensitivity of detecting corticomotoneuronal dysfunction, by circumventing MEP desynchronization and amplitude variability [127,147,148,149]. TST consists of three successive stimuli delivered at precisely defined ISIs, with motor responses recorded over intrinsic hand or foot muscles. A suprathreshold TMS stimulus (1st TST stimulus) is delivered over the motor cortex, followed by the first electrical stimulus (2nd TST stimulus) delivered over the distal peripheral nerve segment and a third electrical stimulus (3rd TST stimulus) delivered proximally (Erb’s point or gluteal fold). The TMS-induced descending volley collides with the 2nd antidromic stimulus leading to phase cancellation. Subsequently, the 3rd stimulus elicits a supramaximal compound muscle action potential (CMAP) response. The CMAP amplitude and area are compared to a conditioned TST paradigm (without TMS stimulus), resulting in amplitude and area ratios that estimate the proportion of activated corticomotoneurons [147,150,151,152]. Dysfunction of corticospinal tracts is reflected by a reduction in CMAP amplitude and/or area ratios.

*In ALS*, reduction in TST amplitude ratio is a sensitive physiological biomarker of corticomotoneuronal dysfunction, evident in 62% of the patients and is more sensitive than MEP reduction or CMCT prolongation [153]. At a pathophysiological level, reduction in TST amplitude ratio correlates with functional disability and UMN dysfunction. Additionally, abnormalities of TST amplitude and area ratios have also identified subclinical UMN dysfunction [154,155,156,157], implying diagnostic utility. Given the commercialization of TST software, translation into clinical practise as a surrogate diagnostic test is now possible.

## 5. Resting State-EEG

Electroencephalography (EEG) captures the projected activity of underlying inhibitory and excitatory neuronal populations by recording the electrical field passing between the originating neuronal activity and the sensor. EEG spatial resolution is determined by the number of recording electrodes—the international 10–20 system, commonly used in clinical practise, defines the position of 21 electrodes and higher density variants, the 10–10 and 10–5 systems, use 64 and 128 electrodes respectively. EEG signals are commonly divided into 5 major frequency bands, namely, delta (δ) 0.1–4 Hz, theta (θ) 4–8 Hz, alpha (α) 8–12 Hz, beta (β) 12–30 Hz, and gamma (γ) > 30 Hz and quantitative EEG measures have traditionally examined power spectra within these frequency bands. Advances in technology have improved the source localisation of neuronal activity. This, concurrent with the exquisite temporal resolution of EEG has enabled the development of functional resting state connectivity models and the identification of EEG microstates.

Early EEG recordings in ALS patients identified reductions in alpha power over the sensorimotor cortex [158,159] with changes attributed to the process of neurodegeneration. Alpha power captured by EEG is thought to reflect the preponderance of inhibitory cortical activity [160] and, as such, findings of reduced sensorimotor alpha power in ALS patients are concordant with the presence of motor cortex hyper-excitability.

Employing a network approach, simple connectivity analyses of resting state EEG demonstrated increased connectivity in fronto-central regions in early-stage of ALS [161]. Specifically, disruption to the *Salience and Default Mode* networks is evident in the early stages of ALS [161]. An increased complexity of resting state networks was concurrently evident, reflecting pathological re-organisation [161].

Separately, motor activity is associated with changes in *β* frequency oscillations. While the physiological mechanisms remain to be fully elucidated, movement initiation is associated with a drop in β-band power (referred to as desynchronisation), with β-band power restored at the termination of movement [162]. Within the resting state EEG, sensorimotor network β-band power is significantly reduced in ALS, and this reduction correlates with motor disability and disease progression [163].

The phenotypic heterogeneity evident in ALS patients has led to a search for neurophysiological biomarkers that could identify reliable cortical signatures [164]. Utilizing resting state EEG with sophisticated signal analysis, specific electrical cortical patterns have bene identified including: (i) *α-band synchrony* [the disruption of somatomotor networks]; (ii) *β-band neural activity* [the disruption of fronto-temporal networks], and (iii) *γ1-band synchrony/co-modulation* [the disruption of fronto-parietal networks]. While resting state-EEG offers promise in ALS research, potentially providing novel insights into cortical neuronal network dysfunction, particularly in the setting of motor cortex inexcitability or marked LMN degeneration precluding the recoding of MEP responses, this technique needs further refinement for wider applicability. Specifically, the development of commercially available devices with automated and more precise analytical techniques would assist with the implementation of resting-state EEG in a clinical setting.

## 6. Clinical Utility Provided by Physiological Biomarkers of UMN Dysfunction

**Figure 3:**
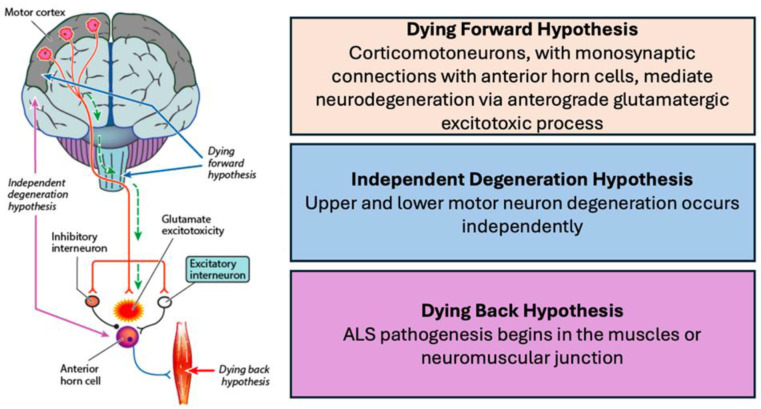
The dying-forward hypothesis proposes that ALS begins centrally and cause anterograde degeneration of bulbar and spinal motor neurons. The independent degeneration hypothesis postulates that upper motor neuron and lower motor neuron dysfunction occur independently. The dying back hypothesis postulates that neurodegeneration originates in peripheral tissues including skeletal muscle and peripheral nerves. Research suggests that the dying-forward hypothesis is the likely mechanism driving ALS.

*Pathogenic insights:* Physiological biomarkers of UMN dysfunction have provided novel and unique insights into ALS pathogenesis. Corticomotoneuronal hyperexcitability was proposed as a pathogenic mechanism in ALS, mediating LMN degeneration via an anterograde glutaminergic process, the “*dying forward hypothesis*” (Figure 3) [10]. Threshold tracking TMS has provided support for the dying-forward mechanism, whereby TMS measures of cortical hyperexcitability (reduced SICI and increased MEP amplitude) correlate with biomarkers of LMN dysfunction/degeneration [51,57,75], precede LMN degeneration [51,165], are associated with specific clinical features [66,67], and patterns of disease evolution [55,59]. Additionally, cortical hyperexcitability is associated with a faster rate of progression, greater functional disability, and reduced survival [61,104,166]. Concurrently, increased activity of high threshold facilitatory interneuronal networks (reflected by increased SICF) is evident in ALS and correlates with a greater level of UMN dysfunction and functional decline. The presence of cortical hyperexcitability reliably differentiates ALS from neuromuscular mimicking disorders [28], thereby arguing against the notion of a plasticity phenomenon.

Cortical hyperexcitability may represent a crucial step prior to the onset of LMN degeneration in the multistep model of ALS [64,65,86]. It should be stressed that ALS is a complex neurodegenerative disorder, mediated by genetic, molecular, and environmental mechanisms. At a molecular level, glutamate excitotoxicity, oxidative stress, autophagy, impaired RNA and lipid metabolism, mitochondrial dysfunction, impaired axonal transport, aberrant neuroinflammation, and proteasome dysfunction have all been implicated in ALS pathogenesis [4,167]. Protein aggregation, secondary to specific ALS genetic mutations further contribute to pathogenesis. Whether the development of cortical hyperexcitability is an initiating event or develops in the final stages of the ALS pathogenic multi-step process needs to be clarified.

It could be argued that corticomotoneuronal hyperexcitability may be a conduit for disease evolution and clinical heterogeneity evident in ALS [18]. A contiguous horizontal evolution of disease (limb-to-limb) is most frequently reported in ALS [104,168,169], with concordance between UMN and LMN dysfunction in the affected body region, and between disease onset site onset and limb dominance [170,171]. A potential explanation for this concordance may relate to a higher density of corticospinal tract innervation [172], and thereby more prominent corticomotoneuronal hyperexcitability in the affected region. The variability in disease spread and clinical heterogeneity may relate to the proclivity of specific corticomotoneuronal networks for the development of hyperexcitability. Support for this notion is provided by TMS studies disclosing focal onset of cortical hyperexcitability in the motor cortex corresponding to disease onset site [59,104], as well as transgenic mouse models establishing that mis-localisation of TDP-43 to UMNs increases excitatory inputs to spinal motor neurons and induces disease progression through a dying forward mechanism [173].

*Diagnostic utility:* In the absence of a pathognomonic test, the diagnosis of ALS relies on identifying progressive clinical upper and lower motor neuron signs and exclusion of potential mimicking disorders [1,6,167,174]. Clinical and neuropsychological-based diagnostic criteria have been developed to facilitate an earlier and more definitive ALS diagnosis [6,7,8,175], critical for guiding clinical management and recruitment into clinical trials. The El-Escorial revised, and Awaji-Shima criteria are complex with multiple levels of diagnostic certainty (*definite, probable, possible*) [6,7,8,175]. The complexity of criteria and reliance on clinical assessment of UMN dysfunction has resulted in suboptimal sensitivity as well as poor-interrater reproducibility [176,177,178,179]. The Gold Coast criteria was recently proposed [6], with diagnostic certainty levels excluded and the presence of UMN and LMN dysfunction in one body region, or LMN dysfunction in two regions, deemed diagnostic of ALS in the setting of disease progression and exclusion of mimicking disorders. While the Gold Coast criteria exhibited increased sensitivity and a comparable specificity when contrasted with the previous criteria [180], the identification of UMN dysfunction remained clinically based, which may be difficult in the setting of LMN dysfunction [181].

Reduction in SICI, as measured by the threshold tracking TMS technique (serial ascending Sydney method) [35], was shown to be a robust and objective biomarker of UMN dysfunction in ALS [28,143], improving the diagnostic utility of the Awaji criteria by ~34% [28] and hastening diagnosis by ~8 months [143]. The utility of threshold tracking TMS has been of utility in lower motor neuron predominant phenotypes, where the detection of sub-clinical UMN dysfunction has aided diagnosis [56,75]. This finding has been re-affirmed by studies that implemented the parallel threshold tracking method [53,54]. The commercialisation of threshold-tracking software and integration into commercially available neurophysiological devices will further clarify the utility of SICI as a diagnostic physiological biomarker.

As discussed above, reduction in TST amplitude and area ratios may be of diagnostic utility in ALS, with sensitivity varying between 54% and 100% [154,155,156,157]. Additionally, TST may help differentiate ALS from mimicking diseases by detecting proximal conduction block as evidenced by multifocal motor neuropathy [182,183], Guillain–Barré syndrome [184] and chronic inflammatory demyelinating polyradiculoneuropathy [185]. The limitations of TST include confounding effects of sub-maximal peripheral stimulation [186] as well as patient tolerability (mainly related to patient discomfort), complexity of the test, and restriction to assessment of intrinsic hand and foot muscles [127,147,148,150]. The absence of a marked reduction in CMAP responses, which typically occur in the later disease stages of ALS, may further limit the diagnostic utility of the TMS techniques.

*Translation to clinical trials:* TMS measures have also shown promise as potential outcome biomarkers in clinical trials [167]. Given the limitations of current clinical measures, there is an urgent need to implement objective outcome biomarkers as primary endpoints in ALS clinical trials [167,174]. Short interval intracortical inhibition appears to be a promising outcome biomarker in clinical trials, as recently demonstrated in trials assessing the effectiveness of retigabine (K^+^ channel modulator) [29] and mexiletine (Na^+^ channel blocker) [187]. Given that SICI reduction has been established as an adverse prognostic factor in ALS [61], and that it is modulated by riluzole (an anti-glutaminergic agent used in the treatment of ALS) [77], the utility of SICI as a biomarker for patient stratification, in addition to an outcome measure, should be considered in future clinical trials.

## 7. Limitations

While the clinical utility of TMS has been reported in multiple previous studies, these have been conducted by a small number of centres. Given the requirement for multiple pieces of equipment, specialised software, and the need for training, the utility of the threshold tracking TMS technique has not been assessed in a multicentre setting. At present, the threshold tracking technique is only available at a few specialised centres, and consequently intrarater and interrater reproducibility and variability, as well as a lack of standardisation across multiple centres limits the translatability of the findings. Recent commercialisation of the threshold tracking methodology and incorporation into an “off-the-shelf” device will enable clarification of its clinical utility. Similarly to threshold tracking TMS, resting state EEG is a complex technique, limited to a few specialised centres, and its utility needs to be established in a multicentre setting, focusing on reliability, reproducibility and standardisation of normative values.

## 8. Conclusions

The abnormalities of threshold-tracking TMS parameters, particularly reduction in SICI, have emerged as important physiological biomarkers of UMN dysfunction in ALS. Specifically, SICI reduction is a robust diagnostic biomarker, reliably differentiating ALS from neuromuscular mimicking disorders. Additionally, the reduction in SICI has been reported as an adverse prognostic biomarker as well as a potentially useful outcome measure in clinical trials. Commercialisation and integration into clinical neurophysiological systems may enhance the wider translation of TMS measures as diagnostic prognostic and outcome measures in neurology.
